# Quartet-based methods to reconstruct phylogenetic networks

**DOI:** 10.1186/1752-0509-8-21

**Published:** 2014-02-20

**Authors:** Jialiang Yang, Stefan Grünewald, Yifei Xu, Xiu-Feng Wan

**Affiliations:** 1 Department of Basic Sciences, College of Veterinary Medicine, Mississippi State University, Mississippi State, MS 39762, USA; 2 Current Address: Department of Genetics and Genomic Sciences, Icahn School of Medicine at Mount Sinai, New York, NY 10029, USA; 3 CAS-MPG Partner Institute for Computational Biology, Key Laboratory of Computational Biology, Shanghai Institutes for Biological Sciences, Chinese Academy of Sciences, Shanghai 200031, China

**Keywords:** Phylogenetic network, Quartet, Reticulate, Reassortant

## Abstract

**Background:**

Phylogenetic networks are employed to visualize evolutionary relationships among a group of nucleotide sequences, genes or species when reticulate events like hybridization, recombination, reassortant and horizontal gene transfer are believed to be involved. In comparison to traditional distance-based methods, quartet-based methods consider more information in the reconstruction process and thus have the potential to be more accurate.

**Results:**

We introduce QuartetSuite, which includes a set of new quartet-based methods, namely QuartetS, QuartetA, and QuartetM, to reconstruct phylogenetic networks from nucleotide sequences. We tested their performances and compared them with other popular methods on two simulated nucleotide sequence data sets: one generated from a tree topology and the other from a complicated evolutionary history containing three reticulate events. We further validated these methods to two real data sets: a bacterial data set consisting of seven concatenated genes of 36 bacterial species and an influenza data set related to recently emerging H7N9 low pathogenic avian influenza viruses in China.

**Conclusion:**

QuartetS, QuartetA, and QuartetM have the potential to accurately reconstruct evolutionary scenarios from simple branching trees to complicated networks containing many reticulate events. These methods could provide insights into the understanding of complicated biological evolutionary processes such as bacterial taxonomy and reassortant of influenza viruses.

## Background

In the natural history of life, recombination, reassortant, hybridization and horizontal gene transfer (HGT) represent four types of important reticulate evolutionary events. For example, recombination plays a significant role in driving human genome evolution [1]; reassortant occurs frequently in influenza viruses and has facilitated the generation of 1957 H2N2, 1968 H3N2, and 2009 H1N1 pandemic influenza viruses [2]; hybridization is crucial in the evolution of plants and fish [3]; and HGT is a significant evolutionary mechanism in shaping the diversification of bacteria genomes [4]. Although most evolutionary events can be modeled as tree-like relationships, these reticulate events can be expressed much more effectively by networks. Moreover, even if the history is not reticulate, parallel evolution, model heterogeneity, and sampling or inference errors may also cause the ambiguity in reconstructing a unique tree. In such cases, networks are efficient in visualizing a group of feasible trees.

Generally speaking, a phylogenetic network is a generalization of a phylogenetic tree, allowing non tree-like structures to represent conflicting signals or alternative evolutionary histories of a group of taxa. Under this umbrella concept, there are two fundamentally different types of phylogenetic networks according to the interpretation of reticulate blocks. An explicit network describes an explicit evolutionary scenario, e.g. hybridization network [3,5], recombination network [6,7] and HGT network [8,9]. In contrast, an implicit network aims to capture incompatibilities in the data, e.g. split network [10,11]. We focus on implicit network in this study.

In past decades, a number of methods have been proposed for reconstructing implicit networks either from pairwise distances, e.g. Split-Decomposition [10,11], Median Network [12], and Neighbor-Net [13] or from weighted triplets and quartets, e.g. QNet [14], SuperQ [15], and QuartetNet [16]. Distance-based methods are usually computationally efficient but tend to be inaccurate since pairwise distances reflect only small information of two taxa, while quartet-based methods are more accurate since quartet contains the information of four taxa. However, simulation studies show that current quartet-based methods are suffered from identifying false-positive and false-negative splits of the taxa set, and the trivial split weights are usually systematically overestimated [16].

Consistency is an important criteria to evaluate the reconstruction performance of a reconstruction method. A reconstruction method is considered as consistent on a special set of trees or networks if the method reconstructs precisely every tree or network in the set provided that the input data are generated from it and that sufficient data are available. Theoretically, one can prove that Neighbor-Joining is consistent on compatible split systems (trees) [17]; Split-Decomposition is consistent on weakly compatible split systems [10,11]; Neighbor-Net, QNet and SuperQ are consistent on circular split systems [13-15]; and Quartet-Net is consistent on 2-weakly compatible split systems [16]. Since 2-weakly compatible split system contains trees, circular split system, and weakly compatible split system as proper subsets, Neighbor-Joining, Neighbor-Net, Split-Decomposition, QNet, and superQ tend to have more false-negatives because these methods restrict the splits they reconstruct to be compatible, weakly compatible, and circular. Any split does not fit the criterion will be removed even if they are true split. Quartet-Net also have the potential to generate false negatives since it takes minimum in calculating the weight of a split whenever there are many possible scenarios [16]. A looser criterion like second minimum, average, or maximum should be able to keep more true splits. In addition, all methods tend to generate some false-positive splits when there are some random mutations or sequence errors, and thus a filtering strategy should be applied to remove the false positives while keeping the final results interpretable.

In this paper, we present three quartet-based methods QuartetS, QuartetA, and QuartetM in QuartetSuite to reconstruct split networks from a collection of weighted triplets and quartets. These methods first calculate triplet and quartet weights directly from multiple sequence alignments (MSAs) by a parsimony method and then functions by iteratively decomposing all triplet and quartet weights into simple components based on full splits. The three methods QuartetS, QuartetA, and QuartetM are designed in the same manner with slight differences. Specifically, QuartetM is a maximum method, in which we take the maximum whenever there are several possible weights of a split. Similarly, QuartetA is an average method and QuartetS is a minimum method. Analyses on simulation data and real data show that these methods are capable of reconstructing accurate phylogenies from branching trees to complicated scenarios containing many reticulate events. In addition, the methods are effective in inferring phylogenetic distances.

## Results and discussion

The proposed quartet-based methods were validated through application into two artificial data and two real data sets. The first artificial data set was simulated from a simple tree phylogeny, whereas the second one was from a complicated phylogenetic scenario containing three reticulate events. The purpose was to show that the quartet-based methods are competent in accurately reconstructing a wide range of phylogenetic networks, from branching trees to very complicated reticulate phylogenies. We chose a bacterial data set consisting of seven concatenated genes of 36 bacterial species whose evolutionary history is generally believed to contain very few reticulate events. We also chose an influenza data set containing 22 selected influenza A viruses related to the evolutionary pathways for the recently emerging H7N9 low pathogenic avian influenza virus. Since the confirmation of the first H7N9 case on March 27, 2013 [18], H7N9 has caused more than 136 human cases in China (http://www.who.int/influenza/human_animal_interface/influenza_h7n9/Data_Reports/en/). The readers are referred to the online website http://sysbio.cvm.msstate.edu/QuartetMethods/ for the nucleotide sequences and nexus files used and generated in this study.

### Simulated data

**Figure 1:**
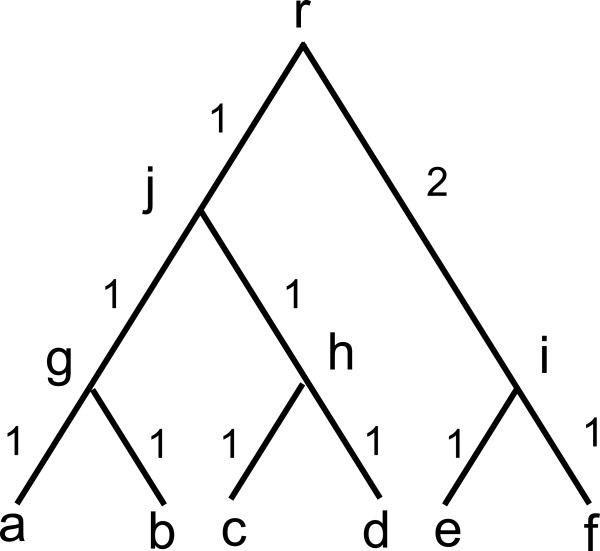
**A phylogenetic tree with 6 leaves.** Each labeled node indicates a taxon and r is the common ancestor. Branch length indicates evolutionary distance between two taxa.

**Figure 2:**
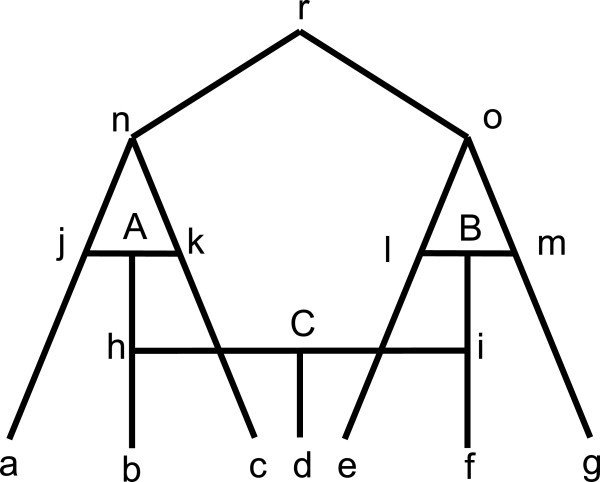
**A phylogenetic network with 3 reticulates.** Each node labeled in lower case letter indicates a taxon and r is the common ancestor. There are three reticulate events labeled by A, B, and C. For example, the sequence of taxa h is resulted from concatenating partial sequence from j and partial sequence from k in reticulate event A.

The software Dawg [19] was applied to generate six DNA sequences from a phylogenetic tree in Figure 1 and seven DNA sequences from a phylogenetic network containing three reticulate events in Figure 2. Specifically, the model is set to be GTR+Gamma+I; the substitution rate is 0.01 and the sequence length is 10,000 bp for tree and 80,000 bp for network since it is a concatenation of eight underlying trees. To avoid randomness, we completed 100 runs of Dawg and the 100 multiple sequence alignment (MSA) were applied to six methods QuartetS, QuartetA, QuartetM, Quartet-Net [16], Neighbor-Net [13], and Neighbor-Joining [20] for phylogenetic tree and network reconstruction.

#### Tree analysis

Table 1 lists all true splits and splits reconstructed by the six methods with bootstrap values larger than or equal to 15 in 100 runs for the tree data. The value in the column “wei” denotes the averaged weight on 100 runs. We only listed one block of a split in Table 1 to increase clarity. For instance, split *ab*|*cedf* is listed as *ab*. We normalized each split with the weight of split *ab* since it was successfully reconstructed by all of these six methods. All methods successfully reconstructed the true splits in all 100 runs. However, Neighbor-Net and Quartet-Net reconstructed a few false-positive splits. For example, Neighbor-Net reconstructed 10 additional splits with bootstrap values varying from 15 to 40. These false-positive splits might be due to some random mutations in the data. In addition, except for QuartetA and QuartetM, all other methods systematically over-estimate the weights of trivial splits and the weights from QuartetA is closer to the true weights with a root mean square error of 0.016. Thus, QuartetA might be good for reconstructing tree-like phylogenies.

**Table 1:** Comparison of true and reconstructed splits and weights on the artificial tree by 6 methods

**True Phylo**		**QuartetS**			**QuartetA**			**QuartetM**			**Quartet-Net**			**Neighbor-Net**			**Neighbor-Joining**		
**Split**	**Wei**	**Split**	**Wei**	**BV**	**Split**	**Wei**	**BV**	**Split**	**Wei**	**BV**	**Split**	**Wei**	**BV**	**Split**	**Wei**	**BV**	**Split**	**Wei**	**BV**
ab	1	ab	1	100	ab	1	100	ab	1	100	ab	1	100	ab	1	100	ab	1	100
abcd	3	abcd	3.03	100	abcd	3	100	abcd	2.98	100	abcd	3.03	100	abcd	2.98	100	abcd	2.98	100
abef	1	abef	1.03	100	abef	1.03	100	abef	1.03	100	abef	1.03	100	abef	1.02	100	abef	1.02	100
a	1	a	1.03	100	a	0.99	100	a	0.98	100	a	1.03	100	a	1.04	100	a	1.04	100
b	1	b	1.04	100	b	1	100	b	0.99	100	b	1.04	100	b	1.05	100	b	1.05	100
c	1	c	1.03	100	c	0.99	100	c	0.98	100	c	1.03	100	c	1.04	100	c	1.04	100
d	1	d	1.03	100	d	0.99	100	d	0.98	100	d	1.03	100	d	1.04	100	d	1.04	100
e	1	e	1.04	100	e	1.01	100	e	1.00	100	e	1.04	100	e	1.04	100	e	1.05	100
f	1	f	1.06	100	f	1.03	100	f	1.02	100	f	1.06	100	f	1.06	100	f	1.06	100
											abc	0.01	15	abc	0.02	31			
											abd	0.02	15	abd	0.02	40			
											acef	0.01	15	acef	0.01	21			
														abf	0.02	34			
														acd	0.02	33			
														aef	0.02	36			
														abe	0.02	35			
														ad	0.01	16			
														ae	0.01	15			
														adef	0.01	19			

The column “True Phylo” denotes the underlying truth and the columns “QuartetS”, “QuartetA”, “QuartetM”, “Quartet-Net”, “Neighbor-Net”, and “Neighbor-Joining” denote the reconstructed results by each method respectively. In addition, “Wei” denotes the average weight of the corresponding split over 100 runs; “BV” denote bootstrap value.

#### Analysis on a phylogenetic network with three reticulate events

In Table 2, all true splits and splits reconstructed by the six methods with bootstrap value larger than or equal to 10 for the network data are listed. The weight of a true split in Figure 2 is calculated as summing up the split weights of this split in eight underlying trees by switching off one branch in a reticulate event. For example, an underlying tree is obtained by switching off three branches jA, hC, and iB in reticulate events A, B, and C. Similarly, we also normalize each split with the weight of split *abc* and then multiple it by four for convenience. As can be seen from the table, QuartetS, QuartetA, QuartetM, and Quartet-Net accurately reconstruct all the true splits in all 100 runs, while Neighbor-Net and Neighbor-Joining fail to reconstruct a lot of true splits. For example, Neighbor-Net fails to reconstruct the split *acefg* in more than 90 out of 100 runs and Neighbor-Joining fails to reconstruct splits *abd*, *abce*, *abcg*, *aefg*, *abceg*, and *acefg* in all 100 runs. This occurs because that Neighbor-Joining only reconstruct trees and Neighbor-Net also reduces the splits to make the split system planar. As a result, the reconstructed trees and networks are much simpler than the true phylogeny, distorting the originally complicated evolutionary histories. In addition, Neighbor-Net and Quartet-Net also reconstruct a few false-positive splits with low weights. It is worth noting that QuartetS reconstructs the closest non-trivial split weights with a root mean square error of 0.054 and QuartetA reconstructs the most accurate trivial split weights with a root mean square error of 0.124. Thus, QuartetS and QuartetA could be useful in reconstructing phylogenetic networks with a lot of reticulate events.

**Table 2:** Comparison of true and reconstructed splits and weights on the artificial network by 6 methods

**True Phylo**		**QuartetS**			**QuartetA**			**QuartetM**			**Quartet-Net**			**Neighbor-Net**			**Neighbor-Joining**		
**Split**	**Wei**	**Split**	**Wei**	**BV**	**Split**	**Wei**	**BV**	**Split**	**Wei**	**BV**	**Split**	**Wei**	**BV**	**Split**	**Wei**	**BV**	**Split**	**Wei**	**BV**
ab	1	ab	1.08	100	ab	1.09	100	ab	1.12	100	ab	1.08	100	ab	0.76	80	ab	0.41	56
abc	4	abc	4	100	abc	4	100	abc	4	100	abc	4	100	abc	4	100	abc	4	100
abd	1	abd	1.03	100	abd	1.03	100	abd	1.03	100	abd	1.02	100	abd	1.32	50			
abcd	4	abcd	3.99	100	abcd	3.99	100	abcd	3.99	100	abcd	3.99	100	abcd	4.17	100	abcd	3.97	100
abce	1	abce	1.01	100	abce	1.02	100	abce	1.01	100	abce	1.01	100	abce	0.86	46			
abcg	1	abcg	1.03	100	abcg	1.03	100	abcg	1.03	100	abcg	1.02	100	abcg	0.89	54			
aefg	1	aefg	1.02	100	aefg	1.03	100	aefg	1.02	100	aefg	1.02	100	aefg	1.39	50			
abcde	1	abcde	1.08	100	abcde	1.09	100	abcde	1.12	100	abcde	1.07	100	abcde	0.76	67	abcde	0.39	46
abcdg	1	abcdg	1.07	100	abcdg	1.08	100	abcdg	1.11	100	abcdg	1.07	100	abcdg	0.71	68	abcdg	0.39	54
abceg	2	abceg	2.07	100	abceg	2.09	100	abceg	2.12	100	abceg	2.07	100	abceg	2.36	47			
acefg	2	acefg	2.06	100	acefg	2.07	100	acefg	2.11	100	acefg	2.06	100	acefg	2.23	8			
adefg	1	adefg	1.08	100	adefg	1.09	100	adefg	1.12	100	adefg	1.07	100	adefg	0.76	73	adefg	0.43	44
a	10	a	10.31	100	a	10.15	100	a	10.17	100	a	10.43	100	a	10.07	100	a	9.42	100
b	6	b	6.25	100	b	6.11	100	b	6.07	100	b	6.33	100	b	7.62	100	b	7.15	100
c	10	c	10.26	100	c	10.11	100	c	10.12	100	c	10.38	100	c	10.05	100	c	9.40	100
d	4	d	4.21	100	d	4.06	100	d	4.08	100	d	4.26	100	d	6.02	100	d	6.82	100
e	10	e	10.28	100	e	10.14	100	e	10.16	100	e	10.41	100	e	10.12	100	e	9.43	100
f	6	f	6.25	100	f	6.11	100	f	6.06	100	f	6.34	100	f	7.22	100	f	7.11	100
g	10	g	10.31	100	g	10.16	100	g	10.18	100	g	10.44	100	g	10.14	100	g	9.47	100
											ac	0.08	99	abdfg	0.04	15			
											abcdf	0.08	97	ag	0.04	13			
											acdeg	0.02	74	ae	0.03	12			
											acdef	0.03	66	abdef	0.04	10			
											ae	0.04	65						
											abdeg	0.03	65						
											ag	0.04	64						
											abdfg	0.04	64						
											acdfg	0.02	62						
											abdef	0.04	59						
											af	0.03	58						

The column “True Phylo” denotes the underlying truth and the columns “QuartetS”, “QuartetA”, “QuartetM”, “Quartet-Net”, “Neighbor-Net”, and “Neighbor-Joining” denote the reconstructed results by each method respectively. In addition, “Wei” denotes the average weight of the corresponding split over 100 runs; “BV” denote bootstrap value.

**Figure 3:**
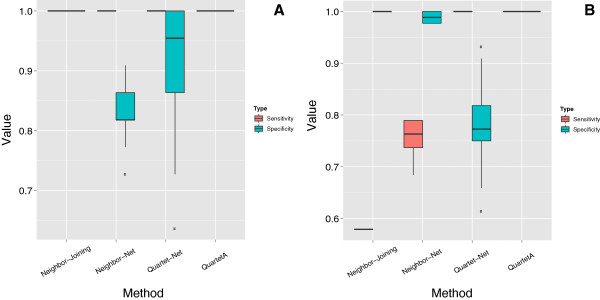
**Comparison of four phylogenetic reconstruction methods in tree (A) and network (B) reconstruction.** The red bar indicates sensitivity and the other one indicates specificity; the horizontal line indicates the median performances.

For a better comparison of the reconstruction performance of QuartetA, Quartet-Net, Neighbor-Net, and Neighbor-Joining on simulated data sets, we plot the sensitivity and specificity of these methods over the 100 runs in Figure 3(A) and 3(B), respectively. We only plot QuartetA because the sensitivity and specificity of QuartetS, QuartetA, and QuartetM are the same by our definition (see Methods). For tree reconstruction, the sensitivities of all methods and the specificities of Neighbor-Joining and QuartetA are equal to 1, while Neighbor-Net has the lowest median specificity. For network reconstruction, only QuartetA has the perfect performance, indicating its potential in reconstructing complex phylogenetic networks, whereas the sensitivity of Neighbor-Joining is the lowest.

### Real data

To remain concise, we only showed the phylogenetic networks constructed by QuartetS as it performs well in reconstructing non-trivial splits in the artificial data above.

#### Analysis on bacteria data

The bacteria sequence data set consists of concatenated sequences of seven important genes (16S rRNA, 23S rRNA, gyrB, phyH, recA, rpoA, and rpoD) from 36 bacteria species, with lengths around 9200 ∼ 12700 base pairs. Each sequence falls into three groups (GC-poor, GC-median, GC-rich) according to their percentage levels (≈30*%*, ≈50*%* and ≈60*%*) of GC content. There are 14 GC-poor, 11 GC-median, and 11 GC-rich bacteria respectively. The readers are referred to [21] for the detailed sequence information of concatenated as well as each gene of the species.

We use Clustal-W [22] to align 11 GC-rich sequences, 25 GC-poor and GC-rich sequences, and all 36 sequences, respectively. The obtained multiple alignments are taken as inputs to QuartetS, QuartetA, QuartetM, Quartet-Net [16], Neighbor-Joining [20], and Neighbor-Net [13]. We ran the program in a Dell desktop with 2.93G HZ processor and 4 GB memory. In practice, Neighbor-joining is the fastest, and the time for QuartetS, QuartetA, QuartetM, and Quartet-Net vary from seconds to around 2 minutes for different MSA sequences. Then, the reconstructed weighted split systems are viewed by SplitsTree [23]. Due to page limitations, we only show the three split networks reconstructed by QuartetS.

**Figure 4:**
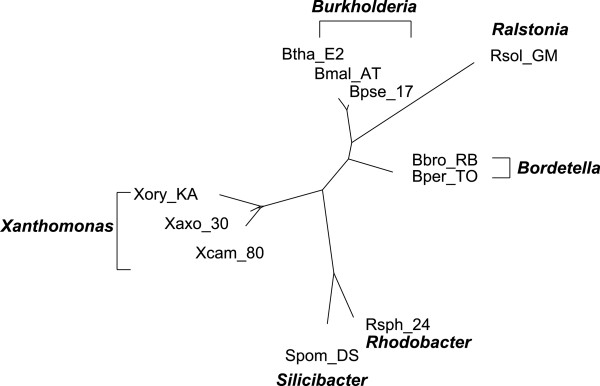
**QuartetS networks on 11 GC-rich bacteria.** Each node indicates a bacteria and the labels in bold font indicate the genus of a group of bacteria.

**Figure 5:**
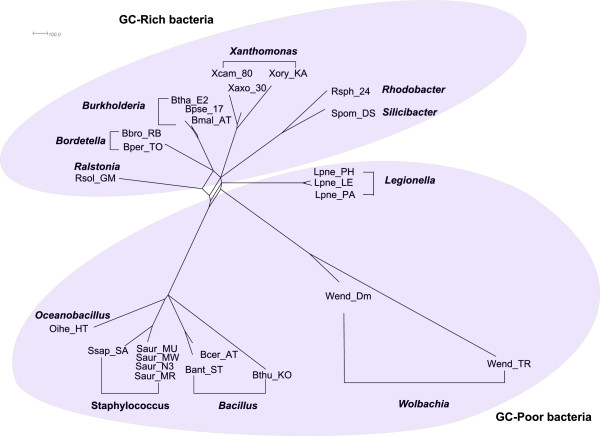
**QuartetS networks on 11 GC-rich and GC-poor bacteria.** Each node indicates a bacteria and the labels in bold font indicate the genus of a group of bacteria. The two groups GC-Rich and GC-Poor bacteria are shaded separately.

**Figure 6:**
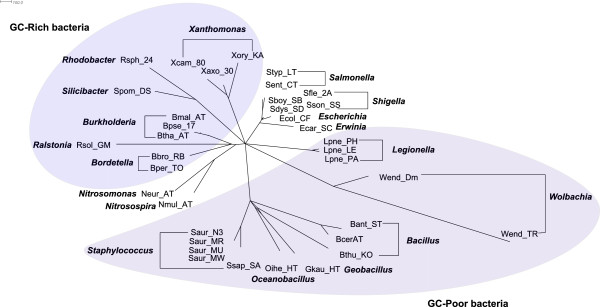
**QuartetS networks on all 36 bacteria.** Each node indicates a bacteria and the labels in bold font indicate the genus of a group of bacteria. The two groups GC-Rich and GC-Poor bacteria are shaded separately.

Figure 4 shows the split network reconstructed by QuartetS on 11 GC-rich bacteria. As commonly believed, it is generally a tree structure with reticulate blocks of very small weights. Figure 5 and Figure 6 show the QuartetS split network on 25 GC-poor and GC-rich bacteria, and all 36 bacteria respectively, which are also generally tree-like with a few reticulation blocks. The bacteria in the same genus are classified together, supporting the current bacterial taxonomy. An interesting observation is that there is a split in Figure 5, which divides the GC-poor and GC-rich bacteria. And the GC-median bacteria are mixed among GC-rich and GC-poor bacteria in Figure 6. The results suggest that GC content might be an important factor in the evolution of bacteria.

#### Analysis on flu data

**Figure 7:**
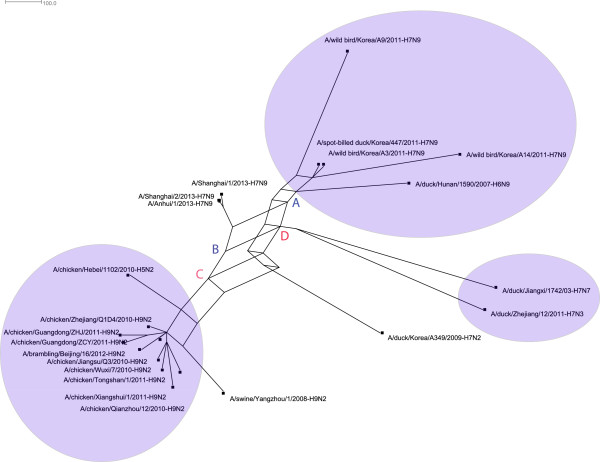
Reassortant network related to H7N9 influenza A viruses.

We downloaded whole genome sequences of 22 influenza A viruses related to the recently emerging H7N9 low pathogenic avian influenza viruses in China [18]. Similarly, we aligned them using Clustal-W [22] and reconstructed the split network by QuartetS. The reconstructed network are shown in Figure 6. In Figure 7, the three viruses that caused human H7N9 cases (A/Shanghai/1/2013, A/Shanghai/2/2013, and A/Anhui/1/2013) are grouped together. The phylogenetic network shows that these viruses were possibly derived from a reassortment event between two ancestral viruses A and B. Virus A might emerge by an reassortment event between avian-origin H7N9 viruses and a virus D associated with H7N3 and H7N7 viruses. Virus B were shown to be genetically linked to H9N2 viruses.

Our results suggested that these novel H7N9 viruses in China are a triple reassortant from avian-origin H7N9 viruses, H9N2 viruses, and H7N3 viruses, which are consistent with other reports [18]. However, the detailed evolutionary process for these H7N9 viruses may be much more complicated, and we will further analyze the details, including the reassortment temporal order and each potential scenario, in the future. A possible direction is to reconstruct the ancestral sequences at the internal nodes by minimizing the number of substitutions. Since there are many methods for ancestral states reconstruction along a tree topology, e.g. [24], we need only reconstruct ancestral states at reticulate blocks, which could be resolved by considering all underlying tree structures and applying parsimony or maximum likelihood criterion at the block.

We have shown that the proposed quartet based methods achieved good sensitivity and specificity on simulated data. However, the comparison of reconstruction methods on real data is hard since the real evolutionary history is unknown. Thus, we only listed the number of full splits reconstructed by QuartetS, QuartetA, QuartetM, Quartet-Net, Neighbor-Net, and Neighbor-Joining in Table 3. It is interesting that sometimes quartet-based methods reconstructed even fewer splits than Neighbor-Joining, indicating that the full resolution of taxa is not achieved. This scenario also happens for other reconstruction methods, e.g. Split-Decomposition [16]. Generally, QuartetS and QuartetM reconstruct very few number of full splits, while QuartetA reconstructs moderate number among the comparison methods.

**Table 3:** Comparison of the numbers of full splits reconstructed by six methods

**Methods**	**GC-Poor**	**GC-Poor and rich**	**All bacteria**	**Influenza**
QuartetS	20	43	56	38
QuartetA	22	59	120	42
QuartetM	21	46	58	40
Quartet-Net	22	45	60	49
Neighbor-Net	29	77	114	68
Neighbor-Joining	19	47	69	41

## Conclusions

We have introduced and implemented three quartet-based methods QuartetS, QuartetA, and QuartetM in QuartetSuite to infer phylogenetic networks from multiple sequence alignment. As can be seen from both simulation data in which the evolutionary histories are known and two real data sets, these three methods can accurately reconstruct phylogenetic scenarios from branching trees to complicated reticulation events. In addition, QuartetS and QuartetM are also good at estimating evolutionary distances between ancestral taxa and current taxa. A comparison study shows that QuartetA is useful in reconstructing tree-like phylogenies, while QuartetS performs well in reconstructing phylogenies with a lot of reticulation events. Our methods have the potential to help untangle the complicated mechanisms underlying evolution.

## Methods

### Splits and their weights

A *split* on a taxa set *X* is a bipartition of *X* into 2 non-empty disjoint subsets (or blocks). The split with two blocks *A* and *B* is denoted by *A*|*B*. If *A* and *B* contain all taxa in *X*, then *A*|*B* is called a *full split*; otherwise, it is called a *partial split*. A split *A*|*B* is called *trivial* if |*A*|=1 or |*B*|=1. For example, the phylogenetic tree in Figure 1 contains six trivial full splits e.g. *a*|*bcdef*, three non-trivial full splits *ab*|*cdef*, *abcd*|*ef*, and *abef*|*cd* and many partial splits, e.g. *ab*|*ef*. For any split *A*|*B*, we define *w*(*A*|*B*) to be the evolutionary distance between taxa set *A* and *B*. For example, *w*(*abcd*|*ef*)=3 in Figure 1. Specifically, if *A*={*a*} and *B*={*b*} contain only 1 taxon in each set, then *w*(*a*|*b*) is the distance between taxon *a* and *b*. A split of type *a*|*bc* is called a *triplet* and thus *w*(*a*|*bc*) is called a triplet weight. Similarly, a split of type *ab*|*cd* is called a *quartet* and *w*(*ab*|*cd*) is called a quartet weight. In general, a split *A*|*B* with |*A*|=*m* and |*B*|=*n* is called an *m*|*n*-split. For any four taxa *a*, *b*, *c*, and *d*, there are three different quartets denoted by *ab*|*cd*, *ac*|*bd*, and *ad*|*bc*, respectively, and thus there are overall 3n4 different quartets for a taxa set of size *n*.

We define a *weighted split system* to be a set of full splits together with their split weights. There is a correspondence between a split network and a weighted split system [10]. For example, a phylogenetic tree defines a natural weighted split system, where each edge in the tree defines a full split and the edge length defines the weight of that split. On the contrary, if all the edges and their lengths are provided, then the tree is fixed [25]. Thus, we can formulate the problem of reconstructing phylogenetic networks as calculating the weight of all full splits for a given taxa set. After the weighted split system is calculated, a software called splitstree [23] is used to visualize the network.

### Calculating triplet and quartet weights

We provide a naive parsimony method to estimate the triplet and quartet weights from the multiple sequence alignment of a taxa set. For any quartet *ab*|*cd*, we first collect the sub-alignment consisting only of the sequences *a*, *b*, *c*, and *d* from the multiple sequence alignment. *w*(*ab*|*cd*) is defined as the proportion of sites such that *a* and *b* share a same character *c*_1_, and *c* and *d* share a character *c*_2_, but *c*_1_≠*c*_2_. This could be considered as a generalization of uncorrected P distance for a pair of taxa. Similarly, one can define triplet weight. It is worth noting that there are a lot of methods to calculate quartet and triplet weight, for example likelihood methods, and the weights from all these methods can be directly applied to our algorithms.

### Quartet based methods

Using the quartet and triplet weights calculated from the multiple sequence alignment, we compute the full split weights from three methods QuartetS, QuartetA, and QuartetM. We used QuartetA as an example and listed the general steps in the following,

**QuartetA:**
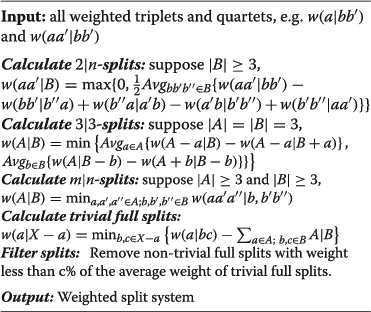


Here, “Avg” means taking the average of all the values. The difference between QuartetS, QuartetA, and QuartetM lies in how the weights of large splits were calculated from those of small ones. In QuartetM, the “Avg” is replaced by maximum value in all possible scenarios, whereas in QuartetS it is the second minimum value.

**Figure 8:**
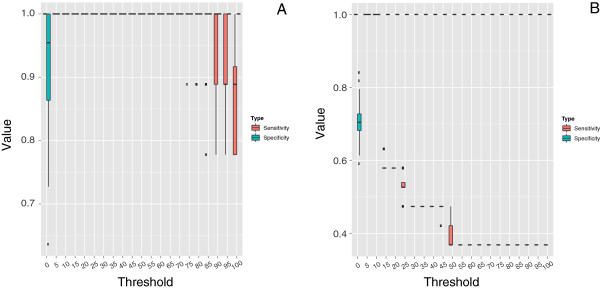
**The influence of threshold*c* on sensitivity and specificity of QuartetS for two simulated data.** The sensitivity and specificity plotted for all 100 runs with the horizontal line in the bar indicating the median performances. The upper whisker extends from the hinge to the highest value that is within 1.5×*IQR* of the hinge, where IQR is the inter-quartile range, or distance between the first and third quartiles. The lower whisker extends from the hinge to the lowest value within 1.5×*IQR* of the hinge. Data beyond the end of the whiskers are outliers and plotted as solid squares.

The parameter *c* is a user defined threshold value for filtering random splits. We test the performance of *c* on two simulated data and the bacteria data. Specifically, we check the variation of sensitivity and specificity of QuartetS by letting *c* vary from 0 to 100 with a step of 5. The results are shown in Figure 8. As can be seen, QuartetS achieves perfect performances when *c* varies from 5 to 70 for tree, and when *c* varies from 5 to 10 for network. Since the simulated tree and network are two extreme cases, we believe that *c*=10 performs well in removing some splits incurred by random mutations and sequencing errors in general. As for bacteria data, we plotted in Additional file 1: Figure S1 the variation of the number of full splits reconstructed by QuaretS and QuartetA with the increasing of *c* since the true evolutionary history is unknown. In Additional file 1: Figure S1, the numbers of full splits reconstructed by both methods decrease with the increase of *c*. *c* being 5 to 10 seems to be able to reduce the complexity of the final network while keeping interpretable results, and all curves becomes flat when *c* is larger than 30. In addition, QuartetA is more affected by the choice of *c* than QuartetS on bacteria data. Theoretically, *c* is related to mutation rate and branch length, and a detailed analysis will be performed in the future.

### Sensitivity and specificity

For *n* taxa, there are overall 2^*n*−1^−1 splits. The splits in the true phylogeny are *condition positives*, and the other splits are *condition negatives*. The splits reconstructed by a reconstruction method are *test outcome positives*, and the other splits are *test outcome negative*. By definition, *true positive* splits are the intersection of condition positives and test outcome positive and *true negative* splits are the intersection of condition negatives and test outcome negative. The *precision* of a reconstruction method is defined as

$$\text{Precision}=\frac{\text{number}\;\text{of}\;\text{true}\;\text{positive}}{\text{number}\;\text{of}\;\text{condition}\;\text{positive}}$$

and *specificity* is defined as

$$\text{Specificity}=\frac{\text{number}\;\text{of}\;\text{true}\;\text{negative}}{\text{number}\;\text{of}\;\text{condition}\;\text{negative}}.$$

#### Implementation

A direct implementation of these methods will lead to exponential algorithms. Similar to [10] and [16], we applied an alternative way that will improve them to polynomial algorithms in most cases. A split *A*|*B* is said to *display* another split *A*^′^|*B*^′^ if either *A*^′^⊆*A* and *B*^′^⊆*B*, or *A*^′^⊆*B* and *B*^′^⊆*A*. For example, *ab*|*cdef* displays *ab*|*cd*. The following lemma is proved in [10].

##### **Lemma1**

If a split *A*|*B* displays another split *A*^′^|*B*^′^, then *w*(*A*|*B*)≤*w*(*A*^′^|*B*^′^).

By this lemma, if a partial split receives weight 0, then all the splits displaying this split will be associated with weight 0. To make use of this property, we implemented quartet methods in the following way: Suppose there are *n* taxa and they are ordered by number 1,2,3,⋯,*n*. There are only three quartets 12|34, 13|24, and 14|23 for the first 4 taxa 1,2,3,4. We stored the quartets together with their weights in an active set, say *S*. After that, iteratively we added *i*=5,6,⋯,*n* to the left and right blocks of the splits stored in *S* and calculated the weights of newly generated splits from those of splits already resolved. Noticing that the only splits that cannot be generated in this way were *ki*|1⋯*k*−1 *k*+1⋯*i*−1 for *k*=1,⋯,*i*−1, we also calculated their weights and added them to *S*. At the end of each iteration, we removed the splits with weight 0 from *S* since they cannot be further extended to splits with positive weights. After the last iteration, only full non-trivial splits with nonzero weights were left in *S*. We also calculated the trivial splits and added to *S*. The process is illustrated in the following algorithm,

##### An alternative way to implementquartet methods

It could be proven that for some special split systems the number of non-zero splits in each iteration are bounded [10,16]. Thus, the time complexity of quartet methods is polynomial for these special split systems like the split system from trees. However, it is beyond the scope of this study. We implemented the algorithm in C++ and the codes are downloadable for free from http://sysbio.cvm.msstate.edu/QuartetMethods/.

## Competing interests

The authors declare that they have no competing interests.

## Authors’ contributions

XW, SG and JY proposed the study; JY and YX performed the experiment; JY and XW analyzed the data and wrote the draft; all authors have read and confirmed the final draft.

## Supplementary Material

Additional file 1 The effect of c on bacteria data. Click here for file

## References

[B1] Meunier J Duret L **Recombination drives the evolution of gc-content in the human genome** Mol Biol Evol 2004 21 6 984 990 10.1093/molbev/msh070 14963104

[B2] Nelson M Vibound C Simonsen L Bennett R Griesemer S George K Taylor J Spiro D Sengamalay NA Ghedin E Taubenberger J Holmes E **Multiple reassortment events in the evolutionary history of h1n1 influenza a virus since 1918** PLOS Pathog 2008 4 2 1000012 10.1371/journal.ppat.1000012 PMC2262849 18463694

[B3] Linder R Rieseberg L **Reconstructing patterns of reticulate evolution in plants** Am J Bot 2004 91 1700 1708 10.3732/ajb.91.10.1700 PMC2493047 18677414

[B4] Doolittle W **How big is the iceberg of which organellar genes in nuclear genomes are but the tip?** Phil Trans R Soc Lond B Biol Sci 2003 358 39 57 10.1098/rstb.2002.1185 12594917 PMC1693099

[B5] Yu Y Than C Degnan J Nakhleh L **Coalescent histories on phylogenetic networks and detection of hybridization despite lineage sorting** Syst Biol 2011 60 2 138 149 10.1093/sysbio/syq084 21248369 PMC3167682

[B6] Gusfield D Eddhu S Langley C **Optimal, efficient reconstruction of phylogenetic networks with constrained recombination** J Bioinform Comput Biol 2004 2 173 213 10.1142/S0219720004000521 15272438

[B7] Huson D Kloepper T **Computing recombination networks from binary sequences** Bioinformatics 2005 21 Suppl 2 159 165 10.1093/bioinformatics/bti1126 16204096

[B8] Kunin V Goldovsky L Darzentas N Ouzounis C **The net of life: reconstructing the microbial phylogenetic network** Genome Res 2005 15 7 954 959 10.1101/gr.3666505 15965028 PMC1172039

[B9] Jin G Nakhleh L Snir S Tuller T **Efficient parsimony-based methods for phylogenetic network reconstruction** Bioinformatics 2006 23 123 128 10.1093/bioinformatics/btl313 17237079

[B10] Bandelt H Dress A **A canonical decomposition theory for metrics on a finite set** Adv Math 1992 92 47 105 10.1016/0001-8708(92)90061-O

[B11] Bandelt H Dress A **Split decomposition: a new and useful approach to phylogenetic analysis of distance data** Mol Phylogenet Evol 1992 1 242 252 10.1016/1055-7903(92)90021-8 1342941

[B12] Bandelt H Forster P Röhl A **Median-joining networks for inferring intraspecific phylogenies** Mol Biol Evol 1990 16 37 48 10331250 10.1093/oxfordjournals.molbev.a026036

[B13] Bryant D Moulton V **Neighbor-net: an agglomerative method for the construction of phylogenetic networks** Mol Biol Evol 2004 21 255 265 14660700 10.1093/molbev/msh018

[B14] Grünewald S Forslund K Dress A Moulton V **Qnet: an agglomerative method for the construction of phylogenetic networks from weighted quartets** Mol Biol Evol 2006 24 532 538 10.1093/molbev/msl180 17119010

[B15] Grünewald S Spillner A Bastkowski S Bogershausen A Moulton V **Superq: Computing supernetworks from quartets** IEEE/ACM Trans Comput Biol Bioinform 2013, in press 10.1109/TCBB.2013.8 23702551

[B16] Yang J Grünewald S Wan X **Quartet-net: a quartet-based method to reconstruct phylogenetic networks** Mol Biol Evol 2013, in press 10.1093/molbev/mst040 PMC3670728 23493256

[B17] Gascuel O Steel M **Neighbor-joining revealed** Mol Biol Evol 2006 23 11 1997 2000 10.1093/molbev/msl072 16877499

[B18] Gao R Cao B Shu Y **Human infection with a novel avian-origin influenza a (h7n9) virus** New England J Med 2013 368 1888 1897 10.1056/NEJMoa1304459 23577628

[B19] Cartwright R **Dna assembly with gaps (dawg): simulating sequence evolution** Bioinformatics 2005 21 31 38 3 suppl 10.1093/bioinformatics/bth471 16306390

[B20] Saitou N Nei M **The neighbor-joining method: a new method for reconstructing phylogenetic trees** Mol Biol Evol 1987 4 406 425 3447015 10.1093/oxfordjournals.molbev.a040454

[B21] Takahashi M Kryukov K Saitou N **Estimation of bacterial species phylogeny through oligonucleotide frequency distances** Genomics 2009 93 525 533 10.1016/j.ygeno.2009.01.009 19442633

[B22] Larkin M Blackshields B Brown N Chenna R McGettigan P McWilliam H Valentin F Wallace I Wilm A Lopez R Thompson J Gibson T Higgins D **Clustal w and clustal x version 2.0** Bioinformatics 2007 23 21 2947 2948 10.1093/bioinformatics/btm404 17846036

[B23] Huson D Bryant D **Application of phylogenetic networks in evolutionary studies** Mol Biol Evol 2006 23 254 267 16221896 10.1093/molbev/msj030

[B24] Yang Z **Paml 4 a program package for phylogenetic analysis by maximum likelihood** Mol Biol Evol 2007 24 1586 1591 10.1093/molbev/msm088 17483113

[B25] Buneman P Hodson FR, Kendall DG, Tautu P **The recovery of trees from measures of dissimilarity.** Mathematics in the Archaeological and Historical Sciences 1971 Edinburgh University Press: Edinburgh 387 395

