# Supplementary material for: Quartet-based methods to reconstruct phylogenetic networks
Source: BMC Syst Biol. 2014 Feb 20;8:21. doi: 10.1186/1752-0509-8-21 (PMC3941989; doi:10.1186/1752-0509-8-21)
Supplement: Additional file 1 — The effect of c on bacteria data. [file 1752-0509-8-21-S1.pdf]

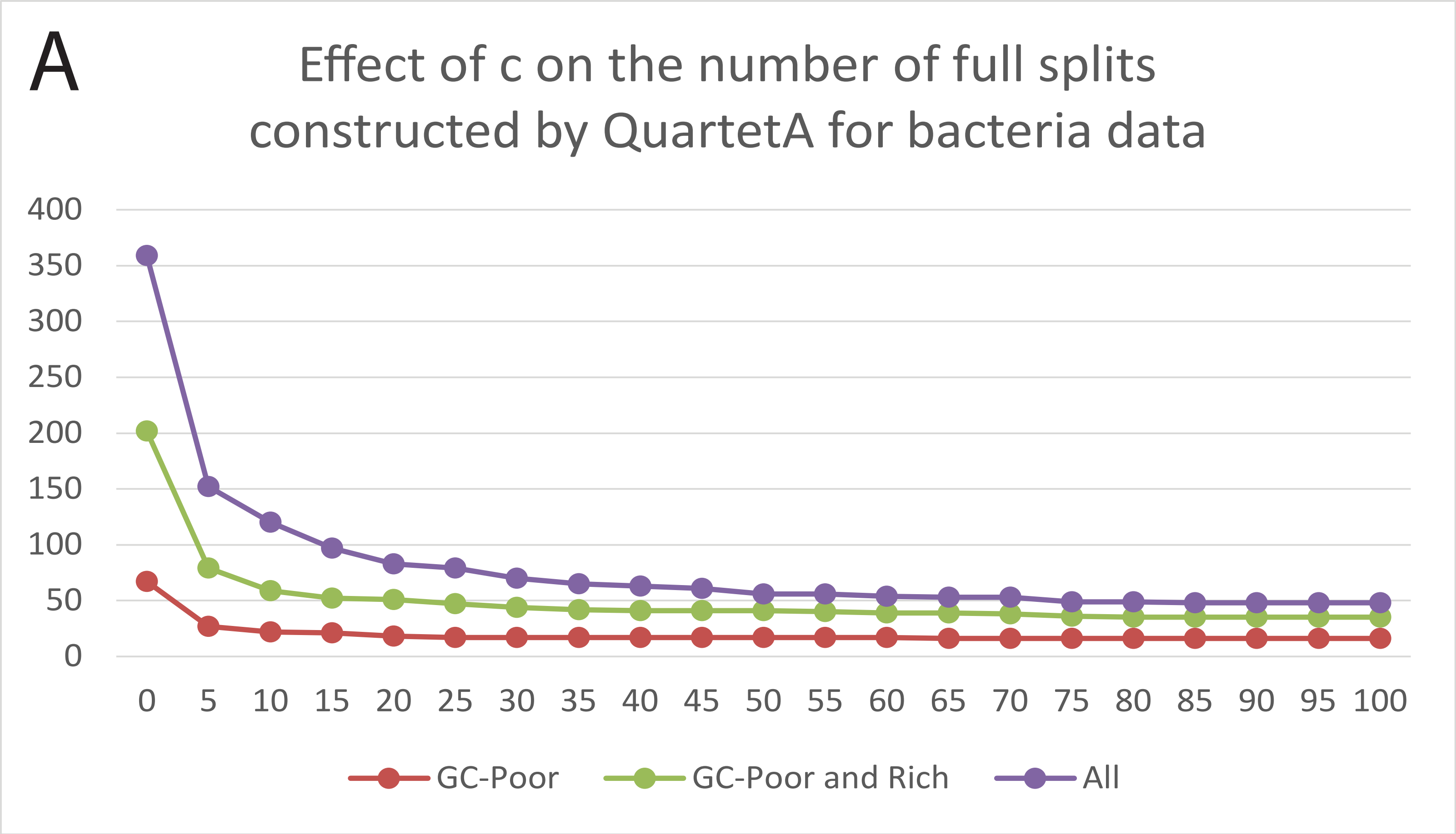
A
Effect of c on the number of full splits constructed by QuartetA for bacteria data
400
350
300
250
200
150
100
50
0
0 5 10 15 20 25 30 35 40 45 50 55 60 65 70 75 80 85 90 95 100
GC-Poor
GC-Poor and Rich
All

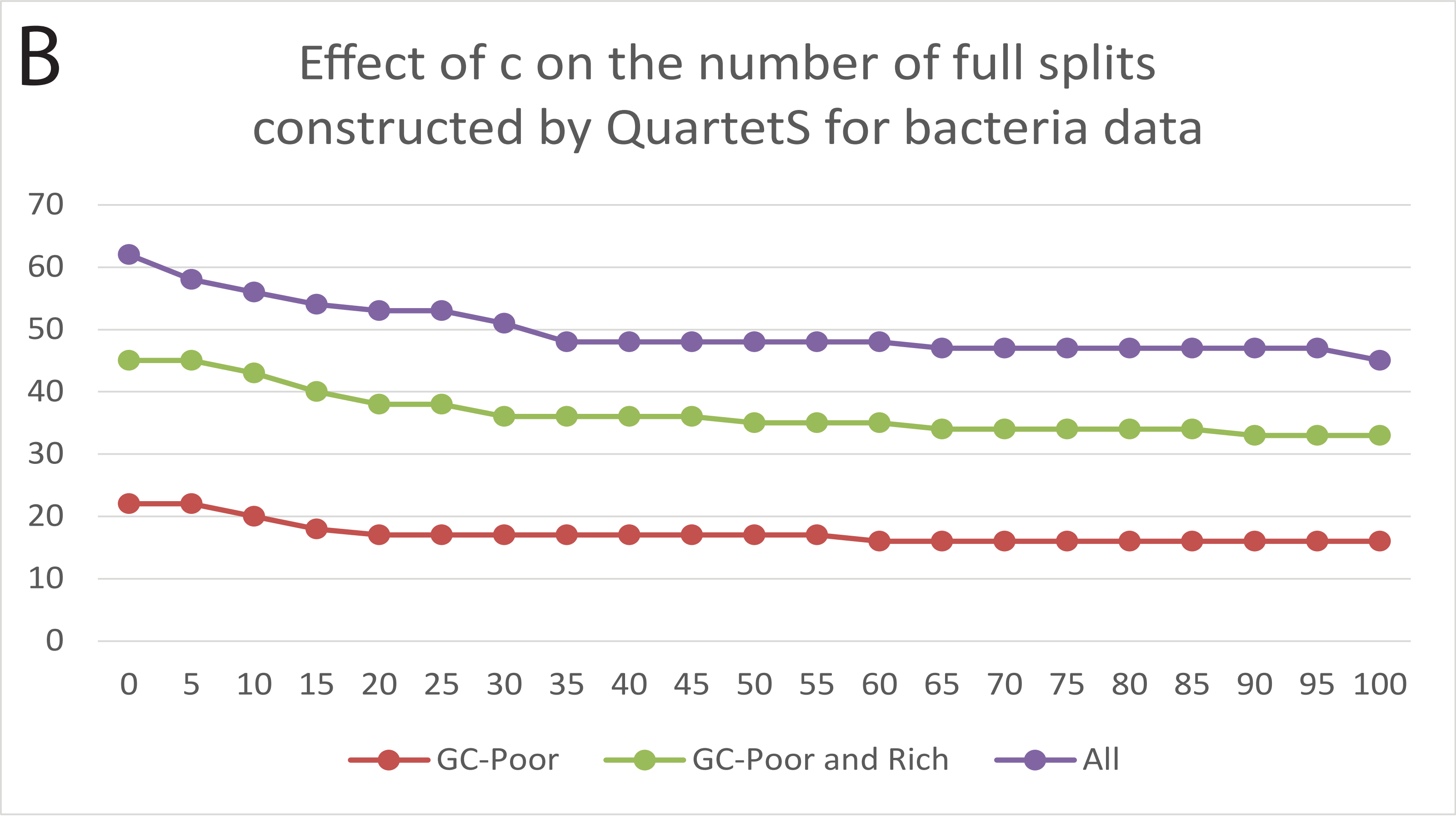
B
Effect of c on the number of full splits constructed by QuartetS for bacteria data
70
60
50
40
30
20
10
0
0 5 10 15 20 25 30 35 40 45 50 55 60 65 70 75 80 85 90 95 100
GC-Poor
GC-Poor and Rich
All
